# Moral injury, perceived burdensomeness, thwarted belongingness, and suicidal ideation in post-9/11 US combat veterans

**DOI:** 10.1080/20008066.2026.2745501

**Published:** 2026-11-03

**Authors:** Sheila F. O’Brien, John David Coppin, Emma Harris, Marcus G. Wild, Alison Krauss, Suzannah Creech

**Affiliations:** a Veterans Integrated Service Network (VISN) 17 Center of Excellence for Research on Returning War Veterans, Waco, TX, USA; b Central Texas Veterans Health Care System (CTVHCS), Temple, TX, USA; c Dell Medical School, University of Texas, Austin, TX, USA; d Central Texas Veterans Research Foundation (CTVRF), Temple, TX, USA

**Keywords:** Moral injury, interpersonal theory of suicide, perceived burdensomeness, thwarted belongingness, suicide, Daño moral, teoría interpersonal del suicidio, percepción de ser una carga, falta de pertenencia, suicidio

## Abstract

**Background:** Moral injury – the psychological harm resulting from *acts of wrongdoing* or *betrayals* – correlates with negative psychosocial outcomes, including increased suicide risk. The Interpersonal Theory of Suicide (IPTS) suggests that *perceived burdensomeness* (the belief that one is a burden to others), and *thwarted belongingness* (psychological pain from unmet social connectedness) confer risk of suicidal ideation.

**Objective:** The current study evaluates the degree to which perceived burdensomeness and thwarted belongingness mediates the associations of acts of wrongdoing and betrayal with current suicidal ideation.

**Method:** We used a generative modelling approach with Bayesian latent variable models in a sample of 312 United States combat-deployed veterans.

**Results:** Acts of wrongdoing were (1) directly associated with current suicidal ideation, with small effect (*β* = 0.15, 95% credible interval (CI): −0.01, 0.30); (2) indirectly associated via perceived burdensomeness, with small effect (*β* = 0.14, 95% CI: 0.06, 0.24); and (3) indirectly associated with thwarted belongingness, with negligible effect (*β* = 0.04, 95% CI: 0.00, 0.10). Acts of betrayal had negligible associations with current suicidal ideation.

**Conclusions:** Feelings of burdensomeness warrant further investigation as a potential clinical target for veterans with moral injury exposure. Thwarted belongingness may be less potent, although measurement error complicates its interpretation.

## HIGHLIGHTS

- We explored the association between potentially morally injurious events and suicidal ideation using a model derived from the Interpersonal Theory of Suicide and we distinguished between acts of wrongdoing- and betrayal-related potentially morally injurious events.
- We tested this mediation model using a generative modelling approach with Bayesian latent variables which accounted for nonnormal and skewed suicidal ideation data.
- We found that acts of wrongdoing were associated with increased suicidal ideation via increased perceived burdensomeness, with small effect, but not via thwarted belongingness.
- Betrayal was not directly or indirectly associated with suicidal ideation.
- Clinical interventions could address a sense of being a burden because of perceived wrongdoing to decrease suicide ideation.

## Abstract (es)

**Antecedentes:** El daño moral – el daño psicológico derivado de actos de transgresión moral o traiciones – se correlaciona con consecuencias psicosociales negativas, incluida un mayor riesgo de suicidio. La Teoría Interpersonal del Suicidio (IPTS, por sus siglas en inglés) sugiere que la percepción de ser una carga (la creencia de que uno supone una carga para los demás) y la falta de pertenencia (el dolor psicológico derivado de la ausencia de conexión social) conllevan un riesgo de ideación suicida.

**Objetivo:** El presente estudio evalúa en qué medida la percepción de ser una carga y la falta de sentido de pertenencia median las asociaciones entre los actos de transgresión moral y la traición; y la ideación suicida actual.

**Método:** Utilizamos un enfoque de modelado generativo con modelos bayesianos de variables latentes en una muestra de 312 veteranos de los Estados Unidos de Norteamérica desplegados en combate.

**Resultados:** Los actos de transgresión moral estuvieron: (1) directamente asociados con la ideación suicida actual, con un efecto pequeño (*β* = 0,15; intervalo de credibilidad [IC] del 95%: −0,01; 0,30); (2) indirectamente asociados a la percepción de ser una carga, con un efecto pequeño (*β* = 0,14; IC del 95%: 0,06; 0,24); y (3) indirectamente asociados con la falta de sentido de pertenencia, con un efecto insignificante (*β* = 0,04; IC del 95%: 0,00; 0,10). Los actos de traición mostraron asociaciones insignificantes con la ideación suicida actual.

**Conclusiones:** Se justifica una mayor investigación en la sensación de ser una carga como posible objetivo clínico para los veteranos expuestos a daño moral. El sentido de pertenencia frustrado podría tener una relevancia menor, aunque el error de medición complica su interpretación.

## 1. Introduction

‘Moral injury’ describes the harm that people may suffer when their deeply held notions of right and wrong are transgressed either by their own actions or inactions (*acts of wrongdoing*) or being directly impacted by another’s actions (*betrayals*); these transgressions are called ‘potentially morally injurious events’ (Griffin et al., 2019; Litz et al., 2022; Williamson et al., 2021). In warzones, potentially morally injurious events may occur because of organizational circumstances (e.g. inaccurate intelligence or poor leadership); environmental circumstances (e.g. persistent chaos and split-second decision-making); cultural and relational circumstances (e.g. dehumanization of the enemy); and psychological circumstances (e.g. emotional numbness and desire for retribution) (Currier et al., 2015). Moral injury impairs functioning through alterations in one’s sense of self and relatedness to others, social behaviour, moral thinking and emotions, beliefs about one’s meaning and purpose, as well as increased self-harm and self-handicapping (Litz & Walker, 2025). Among nationally representative samples of US veterans, reports of potentially morally injurious events were associated with increased odds of suicidal ideation (Wisco et al., 2017) and peri- and post-military suicide attempts (Maguen et al., 2022). Among workers in high stress occupations, such as the military, moral injury ‘caseness’ is associated with substantially higher odds of lifetime suicide attempts (versus PTSD, OR = 1.94; depression OR = 1.19) (Griffin et al., 2025). Although moral injury and potentially morally injurious events seem to increase military and veteran suicide risk, there are few conceptual models or theories that explain why or how moral injury contributes to this risk.

The Interpersonal Theory of Suicide (IPTS; Van Orden et al., 2010), a leading explanatory theory of suicidal ideation and attempts, proposes that a person will engage in suicide behaviors when they possess both a desire to die (operationalized as suicidal ideation with suicide intent) and acquire the capability to enact a suicide behaviour. Within this theory, suicidal ideation and intent develop from a combination of perceived burdensomeness and thwarted belongingness. Perceived burdensomeness is the perception of being a burden to others and a sense of self-hate (e.g. significant shame, guilt, anger). Thwarted belongingness is characterized by loneliness and a lack of reciprocally caring relationships. The IPTS has been applied (Silva et al., 2017; Wolfe-Clark & Bryan, 2017) and adapted (Sokol et al., 2021) to veteran populations in particular, although empirical findings are equivocal. In particular, some evidence supports the role of thwarted belongingness in suicidal ideation (Hom et al., 2017; O’Connor et al., 2017; Rogers et al., 2017) whereas some evidence suggests that perceived burdensomeness, but not thwarted belongingness, predicts suicidal ideation among veterans (Compton et al., 2021; Kolnogorova et al., 2021; Martin et al., 2021).

We speculate that the impact of potentially morally injurious events could partially account for the prior mixed findings on the IPTS in veterans. To date, a handful of studies have examined facets of moral injury in the context of the IPTS. In samples of previously deployed US military personnel, acts of wrongdoing and betrayals were correlated with thwarted belongingness and perceived burdensomeness, with small effects (*r*’s = .15-31; Houtsma et al., 2017). Among female National Guard service members, thwarted belongingness and perceived burdensomeness indirectly accounted for the association between potentially morally injurious events and suicidal ideation, with very small effects (*b* = 0.002, SE = 0.001, 95% CI [0.001, 0.007]; *b* = 0.003, SE = 0.001, 95% CI [0.001, 0.008]; Shapiro et al., 2022).

Overall, the current body of literature on the associations between moral injury and suicide risk is limited because few studies have theory-driven hypotheses based on the well-established IPTS. Few studies examined whether potentially morally injurious events are associated with greater perceived burdensomeness and thwarted belongingness and whether these associations account for a link between potentially morally injurious events and suicidal ideation. Additionally, few studies examined whether the relations between potentially morally injurious events and suicidal ideation differs depending on the facet of potentially morally injurious event (e.g. acts of wrongdoing versus betrayal) and these facets’ associations with perceived burdensomeness and thwarted belongingness.

The current study estimates the effects of the theorized relations between potentially morally injurious events and suicidal ideation within the context of the IPTS. Specifically, we evaluated the degree to which perceived burdensomeness and thwarted belongingness mediates the relation between potentially morally injurious events and suicidal ideation, and we distinguished between acts of wrongdoing and betrayal. First, we expected that potentially morally injurious events would be directly associated with suicidal ideation. Second, we expected that potentially morally injurious events would be associated with greater perceived burdensomeness and thwarted belongingness which, in turn, would be associated with greater suicidal ideation, and that these indirect associations would partially explain the relation between potentially morally injurious events and suicidal ideation.

## 2. Method

### 2.1. Procedures

The present study examines data drawn from the third cohort of a longitudinal study of predictors of and gender differences in family and psychosocial functioning in post-9/11 combat deployed US veterans in the Southwest US (Study Evaluating Returning Veterans Experiences). The present study is a secondary data analysis with aims and research questions that do not overlap with the pre-specified aims of the parent study. The parent study involved four assessments at approximately 8-month intervals (baseline, 8-month, 16-month, 24-month assessments). Current suicide ideation, potentially morally injurious events, perceived burdensomeness, and thwarted belongingness were all assessed at the baseline and 24-month assessments. Thus, we used data from the baseline and 24-month assessments for the current study; data from prior cohorts of the study were not used. Study procedures involved recruiting VA-enrolled veterans via self-referral and letters mailed to potentially eligible veterans identified in VA administrative data. Inclusion criteria were broad: 18-year-old or older men and women with stable psychotropic medications at the time of the baseline. Exclusion criteria were current suicidal or homicidal ideation warranting crisis intervention, mania, psychosis, or severe traumatic brain injury. Current suicide ideation was assessed via interview by study staff; potentially morally injurious events, perceived burdensomeness, and thwarted belongingness was assessed via self-report survey. Participants were compensated $75 each for the baseline and 24-month assessment. See Metts et al. (2025) for a full description of the methods. All participants provided written informed consent prior to participating in the parent study. This study was approved by the Central Texas Veterans Health Care System, Research & Development Committee (that includes the Subcommittee Scientific Review, Subcommittee Research Safety & Institutional Review Board) through 9/30/26 (protocol number #00390).

### 2.2. Participants

The current study active sample was 312 participants. See Table 1 for demographics of sample at baseline.

**Table 1.** Description of sample at baseline.

	Mean/count	*SD/*%
Age	46.40	*9*.*23*
Gender		
Women	83	26.6
Men	229	73.4
Race		
Black/African American	90	28.8
White	172	55.1
Other	50	16.0
Ethnicity (Hispanic/Latino/a/x/e)	53	17.0
Relationship Status*		
Single	61	19.6
Married	224	71.8
Divorced	25	8.0
CSSRS Past 30-days – Time 1		
No Current Ideation	280	89.7
Current Presence of Ideation	25	8.0
Current Presence of Ideation +	7	2.2
CSSRS Past 30-days – Time 2		
No Current Ideation	191	91.0
Current Presence of Ideation	13	6.2
Current Presence of Ideation +	6	2.9

Note. *N* = 312; SD = standard deviation. CSSRS = Columbia-Suicide Severity Rating Scale. *One participant was widowed.

### 2.3. Measures

*Potentially morally injurious events* were assessed using the 9-item Moral Injury Events Scale (MIES; Nash et al., 2013). Items were rated on a Likert-style scale ranging from 1 (*strongly disagree)* to 6 (*strongly agree).* Previous psychometric research suggested that MIES items capture distinct but related acts of wrongdoing and acts of betrayal (Plouffe et al., 2023). Thus, we created two latent factors representing ‘acts of wrongdoing’ (MIES items 1–6) and ‘betrayal’ (MIES items 7–9).

*Perceived burdensomeness* and *thwarted belongingness* were assessed using the 18-item Interpersonal Needs Questionnaire (INQ; Van Orden et al., 2012). Participants respond to items based on current beliefs and feelings using a Likert-style scale ranging from 1 (*not true at all for me*) to 7 (*very true for me)*.

*Current suicidal ideation* was assessed using the Columbia-Suicide Severity Rating Scale (C-SSRS; Posner et al., 2011). The presence of suicidal ideation in the past 30 days was assessed using two binary items (i.e. wishing to be dead, nonspecific suicidal thoughts); we labelled these *Presence 1* and *Presence 2* in Figure 1. If a participant said yes to either item, they were asked four follow-up questions about the *frequency, duration, controllability*, and *deterrents* of their suicidal ideation. Items assessing frequency and duration were rated on a Likert-style scale ranging from 1 to 5; items assessing controllability of thoughts and deterrents to suicide behaviors were rated on a non-Likert scale ranging from 0 to 5. Higher scores indicate greater frequency, longer duration, less controllability, and fewer deterrents. If a participant answered no to both presence of suicidal ideation questions, they did not complete these follow-up items; this skip-logic is the typical clinical administration. This skip logic, however, may underestimate within-person suicide risk because people at greater than low risk of suicide may respond ‘no’ to the ideation questions and ‘yes’ to follow-up questions (Giddens et al., 2014; Tabares et al., 2021). Thus, we estimated latent suicidal ideations for all participants (i.e. including participants who answered ‘no’ to both binary items) based on the full model (i.e. responses to the MIES, the INQ, and their estimated relationship to suicidal ideation using all C-SSRS items). In Table 1, participants who endorsed only *Presence 1* or *Presence 2* items were labelled as ‘Current Presence of Suicidal Ideation and participants who endorsed *Presence 1* and *Presence 2* and completed the *frequency, duration, controllability,* and *deterrents* items were labelled as ‘Current Presence of Suicidal Ideation+’.

**Figure 1.**
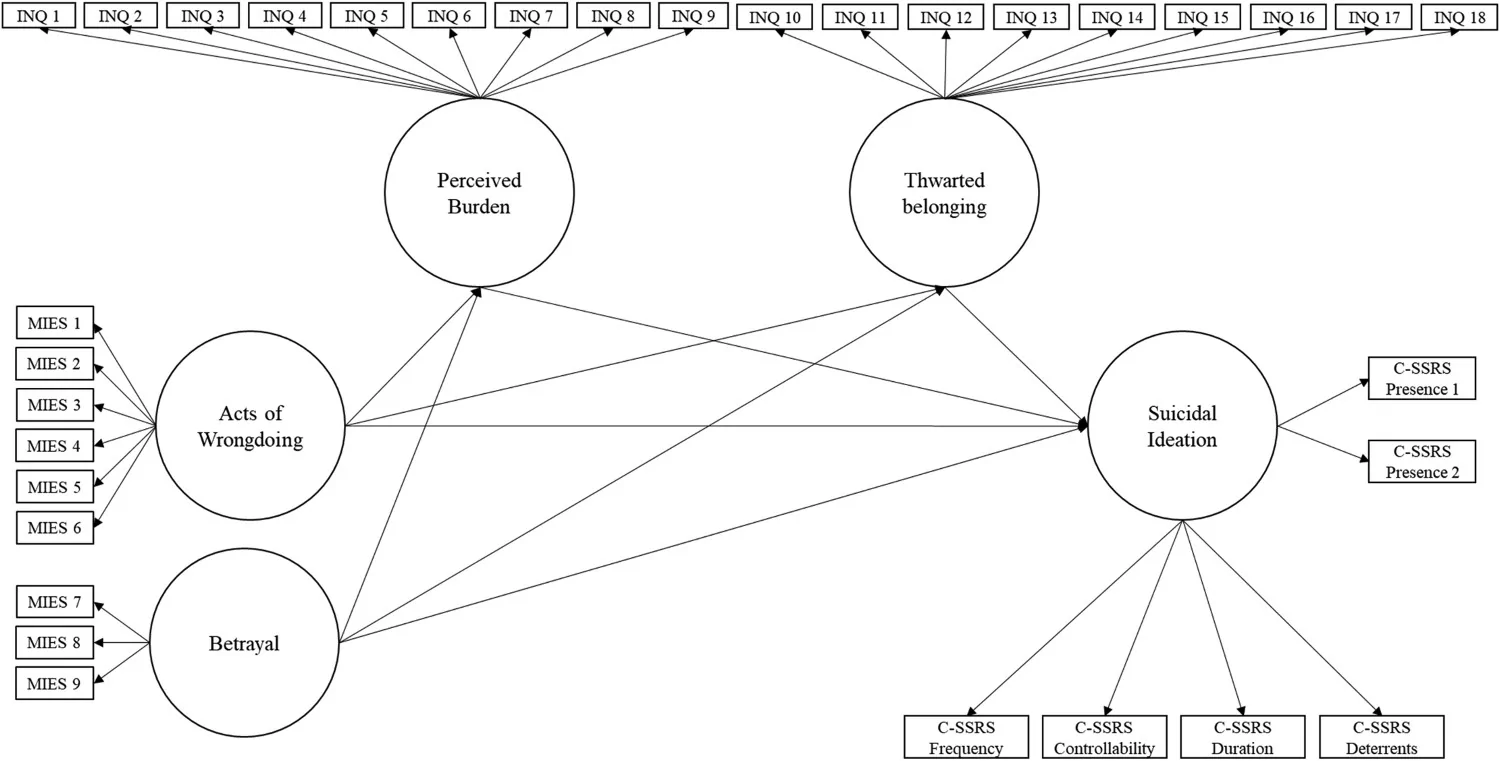
Note: MIES = Moral Injury Events Scale; INQ = Interpersonal Needs Questionnaire; C-SRRS = Columbia Suicide Severity Rating Scale.

### 2.4. Data analysis

#### 2.4.1. Bayesian latent variable generative modelling approach

We used Bayesian latent variable generative modelling to evaluate the degree to which thwarted belongingness and perceived burdensomeness mediated the association between acts of wrongdoing or betrayal and suicidal ideation (Betancourt, 2021). We collated data from the baseline and 24-month assessments to run, essentially, cross-sectional analyses because: (1) the parent study was a longitudinal observational cohort study of a community sample and there was no intervening index event, treatment delivered, or theoretical reason why the variables would have changed; and, (2) suicide is a low base rate event in a community sample. See Figure 1 for the directed acyclic graph (DAG) of the generative model.

A latent continuous variable was mapped to item responses for each measure; binary items were mapped via a Bernoulli model with a probit link function and ordinal Likert-style items were mapped via ordered probit models. The relations among the measures were constructed on the level of the latent continuous variables using normal linear models similar to multiple regression. Prior probability distributions for parameters were evaluated for consistency with domain expertise and logical constraints on the scale of the outcome variables via prior predictive checks. Model fit was evaluated via retrodictive checks, where summary statistics of model posterior predictions are compared to the same summary statistics of the observations. Adequate model fit was ascertained when we saw minimal conflicts between the summary statistics from posterior predictive distributions and from the observed data (Betancourt, 2020; Gabry et al., 2019). See Supplemental material for a conceptual explanation of the model, explanation and plots of all posterior predictive checks, and explanation and plots of all retrodictive checks.

#### 2.4.2. Missing data

Of the 351 veterans in the full sample, 312 provided responses to at least one item within each of the measures of interest and were included in analyses. Of the 312 participants, 165 participants have data at two time points and 147 participants have data at one timepoint. Bayesian methods uses all available data in the sample to estimate a latent variable for each participant. For example, if a participant answered three out of the six C-SSRS items – for instance, the two ideation questions and the controllability question – that participant’s latent suicidal ideation value would be estimated using the information in the those answered items as well as the full joint model for all other measures relating to that latent variable. Bayesian latent variable generative models do not assume normally distributed data and are robust to missingness, which is appropriate for ordinal data on moral injury events and suicidal ideation. These methods do not impute values for missing data.

#### 2.4.3. Statistical software

The model was written and implemented in Stan (Stan Development Team, 2011) via rstan version 2.32.6 in R version 4.4.2 (Stan Development Team, 2024). Stan is a probabilistic programming language that uses Hamiltonian Monte Carlo, a type of MCMC algorithm, for fitting Bayesian models. We fit four chains with 1,000 warmup and 2,000 post-warmup samples. All chains converged as Rhat was less than 1.01 for all parameters. The effective sample size – an estimate of the number of independent samples needed to have the same precision as the MCMC-generated posterior estimates – was greater than 400 for all parameters (>100 samples per chain is usually considered acceptable).

All coefficients for the linear models among the latent variables are reported as standardized to 1 standard deviation. Thus, a coefficient represents the magnitude of change in the latent outcome, in units of standard deviations, given a one standard deviation increase in the latent predictor. To evaluate the findings, we focused on the strength of the effect size (i.e. *β*) rather than on *p*-values or whether the credible interval crossed zero. We interpreted *β* = .10-19 as a small effect, *β* ∼ =  .20–.29 as a medium effect, β ∼ =  .3 as large effect (Nieminen, 2022). We considered *β* < .10 to be a negligible effect and did not interpret these results. Mean and 95% credible intervals (i.e. central 95% quantiles of the posterior distribution, analogous to confidence intervals in frequentist analyses) for the parameters are reported. To better interpret the probability that our findings were meaningful, given that credible intervals may cross zero, we also calculated the probabilities and Monte Carlo standard error that each effect was larger than ≥.10 (i.e. larger than a trivial effect); we reported this as *P*(β≥0.10) in Table 3. Expectation values for the pushforward plots were simulated in R using the vectors of MCMC samples via Monte Carlo simulation. See the supplemental materials for a detailed account of the statistical model, including a brief conceptual description, prior predictive checks, retrodictive checks, and Stan code.

## 3. Results

Table 1 presents descriptive statistics of participant demographics. Table 2 presents descriptive statistics for the predictor variables. Table 3 presents all direct and indirect effects of predictor variables on the outcome variable. The Bayesian latent model indicated a small direct effect of acts of wrongdoing on suicidal ideation such that one standard deviation (SD) increase in latent acts of wrongdoing was associated with an estimated mean 0.15 standard deviation increase in suicidal ideation, with a very strong probability that the indirect effect greater than trivial. We found a small indirect effect of acts of wrongdoing on suicidal ideation through perceived burdensomeness, and a trivial effect of thwarted belongingness on suicidal ideation. We found a trivial direct effect of betrayal on suicidal ideation and trivial indirect effects of betrayal on suicidal ideation through perceived burdensomeness or thwarted belongingness.

**Table 2.** Descriptive statistics of predictor variables.

	Time 1			Time 2		
	*N*	Mean	*SD*	*N*	Mean	*SD*
INQ-18 total	310	48.29	22.72	273	47.40	21.82
Perceived burdensomeness	310	18.83	10.38	273	18.25	9.90
Thwarted belongingness	311	29.38	14.18	273	29.15	13.84
MIES total	311	26.93	12.28	271	24.96	12.10
Wrongdoing	311	17.20	8.50	271	15.70	8.24
Betrayal	311	9.73	5.14	271	9.27	5.13

Note. *SD* = standard deviation. INQ-18 = 18 item version of the Interpersonal Needs Questionnaire. MIES = Moral Injury Events Scale.

**Table 3.** Direct and indirect effects on suicide.

	*β* (95% C.I.)	Probability (*β* > = 0.10) (S.E)
Direct Effect of Wrongdoing on Suicide	0.15 (0.01, 0.30)	0.71 (0.014)
Perceived Burdensomeness	0.14 (0.06, 0.24)	0.81 (0.012)
Thwarted Belongingness	0.04 (0.00, 0.10)	0.03 (0.004)
Direct Effect of Betrayal on Suicide	−0.01(−0.18, 0.16)	0.09 (0.008)
Perceived Burdensomeness	0.09 (0.02, 0.18)	0.39 (0.016)
Thwarted Belongingness	0.06 (0.01, 0.14)	0.13 (0.010)

Note*. N* = 312. C.I. = Credible interval. S.E. = Markov Chain Monte Carlo standard error. ◊ = Indirect effect.

Findings from the mediation model are depicted in Figure 2. When we used the model to simulate responses to individual items, a one SD increase in latent acts of wrongdoing systematically shifted the distributions on items that index thwarted belongingness, perceived burdensomeness, and suicidal ideation. These shifts in the summed score response distributions concord with the estimated relations among the latent variables and depict the shifts towards worsening scores one would expect for this cohort given changes in latent moral injury, albeit with a large amount of variability in the model. We did not create a visualization of the associations between perceived burdensomeness, thwarted belongingness, and suicidal ideation at different levels of betrayal because we found negligible associations between betrayal and suicidal ideation.

**Figure 2.**
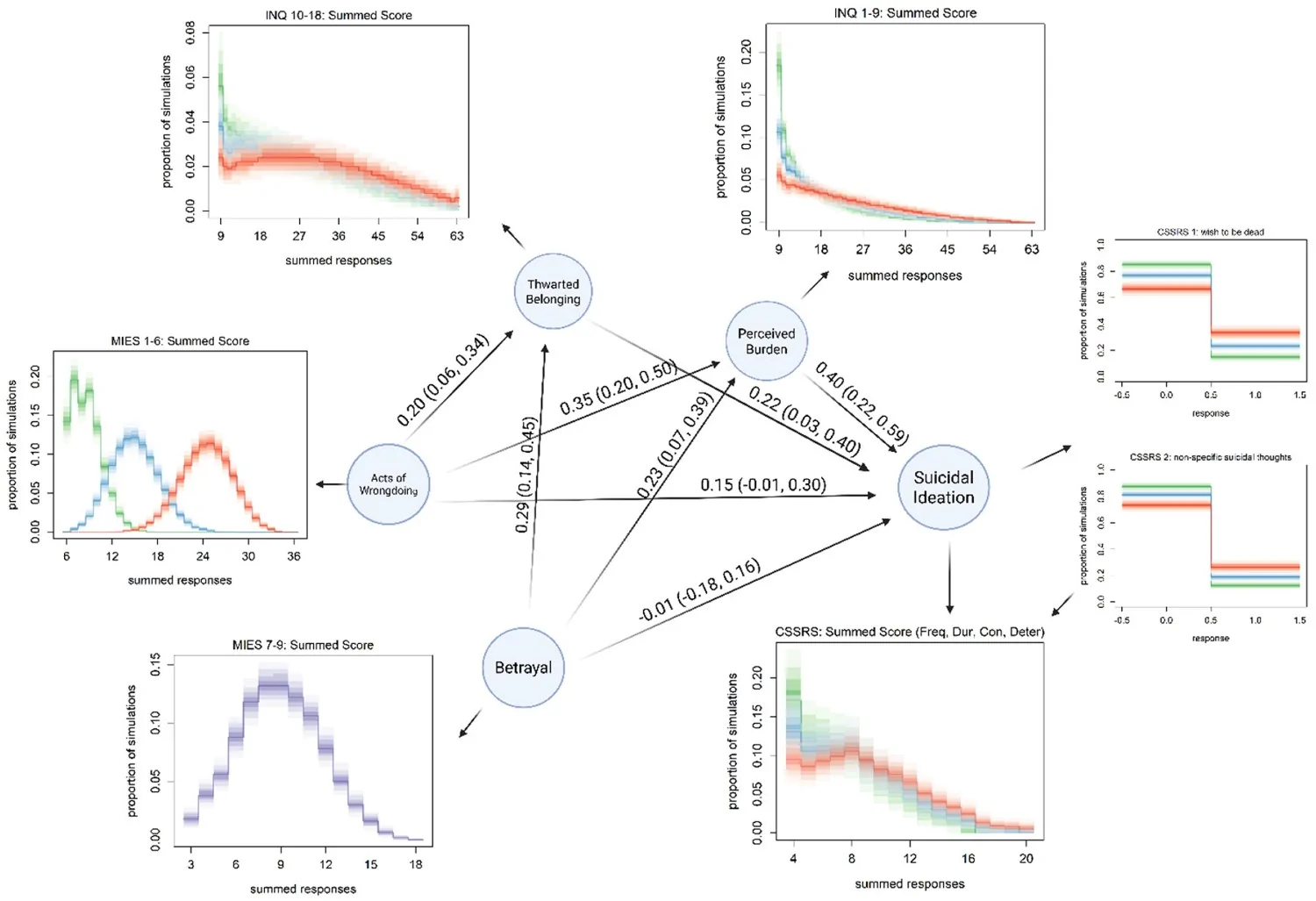
Note: Green lines indicate latent ‘acts of wrongdoing’ set at one standard deviation below its sample mean; blue lines indicate latent ‘acts of wrongdoing’ set at its sample mean; red lines indicate ‘acts of wrongdoing’ set at one standard deviation above its sample mean. These latent ‘acts of wrongdoing’ values are then pushed forward through the model to simulated item responses and subsequently simulated sum scores, resulting in the distributions seen for each measure. Latent ‘betrayal’ is held fixed at its sample mean to show the effect of ‘acts of wrongdoing’. Shading is 10% quantiles from 10-90% quantiles of the Monte Carlo samples. MIES = Moral Injury Events Scale; INQ = Interpersonal Needs Questionnaire; C-SRRS = Columbia Suicide Severity Rating Scale.

## 4. Discussion

Although people who experience potentially morally injurious events are believed to be at higher risk of suicidal ideation (Griffin et al., 2025; Hall et al., 2022), the reasons for this link are not well-tested. We examined whether the IPTS may explain this association using Bayesian latent modelling in a sample of combat-deployed post-9/11 US veterans; we specifically explored the extent to which potentially morally injurious acts of wrongdoing and/or betrayal were indirectly associated with suicidal ideation via associations through perceived burdensomeness and thwarted belongingness. We found that perceived burdensomeness mediated the association between acts of wrongdoing and suicidal ideation, with small effect; thwarted belongingness did so with a trivial effect. Betrayal was associated with suicidal ideation, with a null effect; betrayal was indirectly associated with suicidal ideation via thwarted belongingness and perceived burdensomeness, with negligible effects.

Our results related to perceived burdensomeness align with current conceptualizations of the ‘moral injury’ construct. Specifically, perceived burdensomeness holds aspects of self-hatred and self-blame that map onto moral injury. Shame and guilt following potentially morally injurious events may prompt the sense of self-hatred and self-blame that inheres in perceived burdensomeness. Along these lines, moral injury involves maladaptive meaning making such as seeing oneself as a horrible, bad, or unfit for others’ company because of what one has done or experienced; this maladaptive meaning making could reasonably take the form of beliefs that one is a burden to others. Thus, the construct of perceived burdensomeness may overlap with the construct of moral injury; future research on moral injury and suicide should include tests of extrinsic convergent validity to assess the extent to which facets of moral injury theory and IPTS describe similar or different constructs (Gonzalez et al., 2021).

We found a negligible indirect association between acts of wrongdoing and suicidal ideation via thwarted belongingness. These results were surprising and may be interpreted in light of previous literature on suicide risk as well as prior literature on the measurement of thwarted belongingness. Loneliness and social disconnection (aspects of thwarted belongingness) are established risk factors for suicide (Calati et al., 2019) and may be more potent among those with exposure to PMIEs given the shame, self-hate, and isolation that are characteristic of moral injury. However, although the INQ is the most frequently used measure of perceived burdensomeness and thwarted belongingness, it may not consistently measure the theorized association between thwarted belongingness and suicidal ideation (Hill et al., 2015); thus, findings regarding the thwarted belongingness are interpreted with caution. Thus, the claims made by the IPTS – that thwarted belonginess increases suicide risk – ought to be studied further using alternative methodology in samples of veterans exposed to potentially morally injurious events.

We also did not find a meaningful association between betrayal and suicidal ideation via perceived burdensomeness or thwarted belongingness. Betrayal trauma theory posits that being betrayed by trusted authority figures or caregivers leads to greater distress and memory-related impairments (Smith & Freyd, 2014). For instance, perceptions of institutional betrayal following military sexual trauma were correlated with post-military suicidal ideation and suicide attempts among veteran women (Monteith et al., 2016). Prior research on an association between morally injurious betrayal and aspects of suicide is inconclusive (see Hall et al., 2022 for review). This may be because measures of morally injurious betrayal, in general, do not index the degree of closeness, dependency, or expectations of care between the betrayer and the betrayed.

Our study was marked by a few limitations. Because we leveraged an existing dataset to estimate these associations and because these data were collected prior to the development of measures of moral injury (the psychosocial-spiritual outcome that may follow moral injury events), we could not implement a more robust model that included potentially morally injurious events, IPTS factors, moral injury (the outcome), and suicidal ideation. Although we had data with two timepoints, we did not consider our analyses longitudinal because we did not have three or more time points and so could not adequately model change over time, especially given that some people were missing the second timepoint. A particular strength of our study, however, is that we could also evaluate whether our findings were more likely than chance, by calculating the probability that the effect was larger than trivial.

## 5. Conclusion

The idea that moral injury events contribute to suicide risk has high face validity and great interest in veteran-focused care communities. The IPTS posits several mechanisms explaining the association between moral injury and suicide risk. Findings from this study converge with others highlighting the potential saliency of the acts of wrongdoing and perceived burdensomeness constructs as useful predictors of suicide risk. In the presence of these constructs, clinical work to assist veterans struggling with moral injury could incorporate an understanding of cognitive bias toward perceived burdensomeness into therapy sessions as part of suicide risk reduction efforts. Given that we unexpectedly found that neither morally injurious betrayal nor thwarted belongingness were associated with current suicidal ideation, future research is needed to better understand this divergence from theory. Future research should also examine whether or how moral injury events and/or moral injury can incrementally explain risk of suicidal ideation in the context of other known and established models and risk factors for suicidal ideation and behaviors (Franklin et al., 2017).

## Supplementary Material

Supplemental Materials.docx

## Data Availability

The minimal data set underlying the results described in the manuscript may be made available on request from the corresponding author pending appropriate institutional approval.
