# Supplementary material for: Moral injury, perceived burdensomeness, thwarted belongingness, and suicidal ideation in post-9/11 US combat veterans
Source: Eur J Psychotraumatol. 2026 Nov 3;17(1):2745501. doi: 10.1080/20008066.2026.2745501 (PMC13757814; doi:10.1080/20008066.2026.2745501)
Supplement: Supplementary file 1 [file ZEPT_A_2745501_SM6192.docx]

Supplemental Material Contents

[**Brief conceptual explanation of the model**	2](#_Toc225261339)

[**Prior predictive checks – checking prior modeling assumptions**	8](#_Toc225261340)

[**Retrodictive checks – critiquing model fit**	13](#_Toc225261341)

[**Stan model code**	18](#_Toc225261342)

# **Brief conceptual explanation of the model**

A narratively generative modeling approach seeks to model the probabilistic story of how the data was generated. This is more analogous to ‘mechanistic models’ of the actual process than heuristic style models like regression.

In psychology research, we often wish to measure a latent continuous phenomenon by means of some probe - usually a bank of questions designed to probe the latent phenomenon and provide some quantitative measure of it.

Figure 1 - Ideally, we would directly measure the phenomenon. However, this is usually not possible in psychology where we must rely on quantifying the phenomenon indirectly via probes.

Each probe differs slightly from another and is designed to shed light on a multidimensional phenomenon (construct), but each probe also features error and uncertainty, so the distribution of responses to each probe (item) within a measure may differ.

Since the kinds of probes used are questions (items) that a respondent answers, the observed responses must be discretized. Thus, they are usually in Likert style ordered categories. The distribution of observed ordinal responses to the questions can be thought of as arising from the censoring of the distribution of latent continuous phenomenon. The censoring can be defined by “cut-points” on the latent distribution (Figure 2).

Figure 2 - A latent continuous distribution can be mapped to ordered categorical responses like a Likert scale by dividing the distribution with cut-points (censoring). The difference in the cumulative distribution function (CDF) between cut-points (i.e. area under the curve) corresponds to the probability for the ordered categorical response (the CDF up to the first cut-point and 1-CDF of the last cut-point determine the lowest and highest category probabilities, respectively).

Since the latent continuous phenomenon is unobserved, we a free to choose a mathematically convenient distribution like the standard normal and allow the cut-points to map to the probability for each category in the observed ordered response.

When the latent continuous distribution of the phenomenon is anchored, then the probabilities for each observed ordered category are determined completely by the cut-points’ censoring of the latent phenomenon.

However, when the cut-points are fixed, shifts in the location of the latent distribution perturb the probability of the observed ordered categories by shifting the latent distribution right or left with respect to the fixed cut-points (Figure 3). In this way, larger or smaller values of the latent phenomenon map to higher probability of lower category or higher category responses on the observed ordered categories (Figure 3).

Figure 3 - When the cut-points and the scale of the distribution are fixed, the probabilities of the observed Likert categories are completely determined by the location of the latent distribution. Here, the cut-points and the scale of the distribution remain the same but the location shifts to the right by 1 unit, resulting in a shift to higher probabilities for higher categories of the observed Likert responses.

Varying fixed cut-points by question (probe or item) within the measure (construct) represent the different ways in which the latent phenomenon is censored into the observed ordered categories for each question when the latent continuous phenomenon is fixed across questions (Figure 4). This can be interpreted as representing the differing ways in which each question probes the same latent phenomenon (for example, one question might have high probability of high category responses and another question low probability of high category responses, for the same fixed level of latent phenomenon) (Figure 4). A shift in the latent distribution would perturb the probabilities for each question in similar ways, however, as the same latent distribution (construct or measure) corresponds to each question (item or probe).

Figure 4 - The same latent ‘hungriness’ may be probed in different ways, resulting in different probabilities of the Likert responses for each question, given the same latent distribution of ‘hungriness’. This can be modeled by varying the location of the cut-points for each probe. The same standard normal distribution of latent ‘hungriness’ is shown above, but the two probes have two different sets of cut-points, resulting in different probabilities of the observed Likert responses. In this humorous hypothetical example, this makes sense, as the statement in probe 2, “I feel like eating a burger”, is very particular and may not appeal to everyone like the more general statement “I feel like eating a snack”, given the same level of hunger.

When there are many responses per question, then both the cut-points and the shifts can be inferred for the latent distribution.

In most cases, researchers are interested not in a single phenomenon in isolation, but rather in how different phenomena relate to each other (Figure 5).

Figure 5 - In most cases researchers are interested not in some individual phenomenon but rather in the relationships among many different phenomena

When the probes are questions, this can be thought of as simply an extension of the above-described process where the relationships between phenomena occur on the latent level, and the resulting latent phenomena are then mapped to the observed ordinal responses (Figure 5). The cut-points, shifts, and relationships among the latent variables (phenomenon or measure) can be inferred from the observed Likert responses to each question (item) associated with each phenomenon (measure).

In our analysis, we used Stan’s built-in ordered probit distribution, which models the above process mapping a latent continuous distribution to ordered categories using a standard normal latent distribution. For every measure, such as the 6 items for MIES Wrongdoing, for example, the responses for each item were ordered Likert style responses that were modeled as arising from a censored latent continuous ‘wrongdoing’ distribution. Differing cut-points were inferred for each question. For each person’s observed Likert responses, the shift in latent standard normal distribution was inferred such that it reflected larger or smaller values of latent ‘wrongdoing’ which then mapped to the corresponding probabilities of larger or smaller values on the observed ordered Likert responses. The different phenomena corresponding to the different measures (betrayal, wrongdoing, etc) were related to each other on the latent level, providing the relationships of interest between each latent phenomenon. At the latent level, these relationships were modeled much like usual multiple regression but using the latent phenomenon as the variates and covariates.

The powerful advantage of this modeling approach is that we correctly treat all ordered responses as ordinal and not continuous, whether they are a ‘predictor’ or ‘outcome’ of interest. In addition, since our model is a cohesive framework for the process and all observed responses within a measure arise from a latent continuous distribution, usual obstacles like measures featuring conditional filtering questions or questions with different numbers of categories in the responses, pose no challenges – all responses within a measure, whether binary or Likert, or many questions answered or few answered, arise from the continuous latent variable.

Note that there is a duality between shifting the distribution relative to anchored cut-points and shifting the cut-points relative to an anchored distribution. Most software implements the latter, but it is often easier to conceptualize the former (as described above). For a detailed look at ordinal models with mathematical details see <https://betanalpha.github.io/assets/chapters_html/ordinal_modeling.html> and for the Stan implementation of the ordered_probit_lpmf function see <https://mc-stan.org/docs/functions-reference/bounded_discrete_distributions.html#ordered-probit-distribution>

# **Prior predictive checks – checking prior modeling assumptions**

A Bayesian model consists of a prior model and an observational model (likelihood). The prior model represents the knowledge or domain expertise one has about the model parameters before the data is observed. With any kind of model (Frequentist approaches or Bayesian) assumptions must be made, but it is best practice to check that these assumptions are valid. In a complex model where parameters may be difficult to think about in isolation, the best way to check prior modeling assumptions is via prior predictive checks. Prior predictive checks are done by simulating data from the prior model (the model without the likelihood, i.e. no data) and then summarizing these simulated observations into summaries that are easy to interpret. For example, here we provide prior predictive checks for all items in every measure used in this analysis in the form of histograms.

Each histogram below shows 10-90% quantiles of simulations of Likert responses from the prior model. There is a histogram for every item used in the analysis. These histograms indicate that the priors used in this analysis encode the prior information that Likert responses across all items could vary across the spectrum of ordered responses (relatively non-informative). The slightly higher probability for the lowest or highest category indicates that the latent level relationships among variables are not overly informative but are conservative and regularizing enough to prevent extremely high effect sizes among the relationships between variables (extremes would have manifested as prior predictive histograms with almost all probability in both the first and last categories of the Likert items and relatively little in the middle categories; overly conservative informative priors would have manifested as very little probability in the highest or lowest categories).

***In summary, the histograms below demonstrate that our priors are reasonable, not overly informative, and conservative with regards to effect size.***

# **Retrodictive checks – critiquing model fit**

Proposing, implementing, and fitting what seems like a good generative model is nice in theory but meaningless in practice unless it is checked and critiqued. Retrodictive checking provides a way to critique model fit and suggest necessary changes if needed. Retrodictive checking (aka posterior predictive checking) is accomplished by making predictions of responses from the fitted model, summarizing those predicted responses into some meaningful summary, and then comparing that summary to the same summary of the observed responses (i.e. data). This is ‘retrodicting’ because model predictions are compared to the data used in the analysis, not holdout data. If the model is a good generative model of the process, then it will retrodict the data nicely. Any major discrepancies provide suggestions for a different generative process.

We provide retrodictive checks as histograms of predicted responses compared to histograms of the observed responses for every item used in the analysis. As with the prior predictive checks above, the shades are 10% quantiles from 10-90% quantiles of the Markov Chain Monte Carlo samples predicted responses from the model. Black lines are the histograms of observed responses (i.e. data). Note that the goal is not to ensure that the histogram of observed responses (black lines) falls exactly within each 10-90% quantile (blue shades), but rather to detect any major consistent discrepancies that manifest across the responses or items. There are no such discrepancies here, as the retrodiction is very good across all items. ***This model is a good fit with no retrodictive tension for these summary statistics.***

# **Stan model code**

// this function derives the interior cut-points from the baseline probabilities

functions {

vector make_cutpoints(vector probabilities, int ncuts) {

vector[ncuts] cutpoints;

real running_sum = 0;

for (n in 1:ncuts) {

running_sum += probabilities[n];

cutpoints[n] = inv_Phi(running_sum);

}

return cutpoints;

}

}

data {

int<lower=1> N; // number of latent variables (corresponds to numbers of rows of data when columns are items)

int<lower=1> N_obs_betrayal; // number of observations for each measure (all items within measure combined)

int<lower=1> N_obs_wrongdoing;

int<lower=1> N_obs_belonging;

int<lower=1> N_obs_burden;

int<lower=1> N_obs_si;

int<lower=1> N_obs_sib;

int<lower=1> N_items_betrayal;

int<lower=1> N_items_wrongdoing;

int<lower=1> N_items_belonging;

int<lower=1> N_items_burden;

int<lower=1> N_items_si;

int likert_betrayal[N_obs_betrayal]; // likert responses for all items within a measure

int likert_wrongdoing[N_obs_wrongdoing];

int likert_belonging[N_obs_belonging];

int likert_burden[N_obs_burden];

int likert_si[N_obs_si];

int binary_si[N_obs_sib]; // binary responses for the ideation filtering items

int latent_i_betrayal[N_obs_betrayal]; // indexes for latent variables within each measure

int latent_i_wrongdoing[N_obs_wrongdoing];

int latent_i_belonging[N_obs_belonging];

int latent_i_burden[N_obs_burden];

int latent_i_si[N_obs_si];

int latent_i_sib[N_obs_sib];

int item_i_betrayal[N_obs_betrayal]; // indexes for items within a measure

int item_i_wrongdoing[N_obs_wrongdoing];

int item_i_belonging[N_obs_belonging];

int item_i_burden[N_obs_burden];

int item_i_si[N_obs_si];

int item_i_sib[N_obs_sib];

int<lower=2> ncut_betrayal; // numbers of cut-points for items within each measure

int<lower=2> ncut_wrongdoing;

int<lower=2> ncut_belonging;

int<lower=2> ncut_burden;

int<lower=2> ncut_si;

vector[ncut_betrayal + 1] alpha_betrayal_wrongdoing; // prior for Dirichlet, a vector of c(3,2,1,1,1,1)

vector[ncut_burden + 1] alpha_belonging_burden; // prior for Dirichlet, a vector of c(3,2,2,1,1,1,1)

vector[ncut_si + 1] alpha_si; // prior for Dirichlet, a vector of c(3,2,1,1,1)

}

parameters {

simplex[ncut_betrayal + 1] pi_betrayal[N_items_betrayal]; // baseline probabilities from which cut-points are derived for each item

simplex[ncut_wrongdoing + 1] pi_wrongdoing[N_items_wrongdoing];

simplex[ncut_belonging + 1] pi_belonging[N_items_belonging];

simplex[ncut_burden + 1] pi_burden[N_items_burden];

simplex[ncut_si + 1] pi_si[N_items_si];

vector[N] betrayal; // vectors of latent variables

vector[N] wrongdoing;

vector[N] belonging;

vector[N] burden;

vector[N] ideation;

real alpha_sib; // parameters for the Bernoulli probit model of binary responses to ideation filtering questions

vector<lower=0>[2] b_sib;

real beta_belonging_betrayal; // parameters for latent level models

real beta_belonging_wrongdoing;

real beta_burden_wrongdoing;

real beta_burden_betrayal;

real beta_ideation_betrayal;

real beta_ideation_wrongdoing;

real beta_ideation_burden;

real beta_ideation_belonging;

real<lower=0> sigma_belonging;

real<lower=0> sigma_burden;

real<lower=0> sigma_ideation;

}

// the following block derives the interior cut-points from baseline probabilities

transformed parameters {

vector[ncut_betrayal] cutpoints_betrayal[N_items_betrayal];

vector[ncut_wrongdoing] cutpoints_wrongdoing[N_items_wrongdoing];

vector[ncut_belonging] cutpoints_belonging[N_items_belonging];

vector[ncut_burden] cutpoints_burden[N_items_burden];

vector[ncut_si] cutpoints_si[N_items_si];

for (n in 1:(N_items_betrayal)) {

cutpoints_betrayal[n] = make_cutpoints(pi_betrayal[n], ncut_betrayal);

}

for (n in 1:(N_items_wrongdoing)) {

cutpoints_wrongdoing[n] = make_cutpoints(pi_wrongdoing[n], ncut_wrongdoing);

}

for (n in 1:(N_items_belonging)) {

cutpoints_belonging[n] = make_cutpoints(pi_belonging[n], ncut_belonging);

}

for (n in 1:(N_items_burden)) {

cutpoints_burden[n] = make_cutpoints(pi_burden[n], ncut_burden);

}

for (n in 1:(N_items_si)) {

cutpoints_si[n] = make_cutpoints(pi_si[n], ncut_si);

}

}

model {

// prior model

betrayal ~ normal(0, 1);

wrongdoing ~ normal(0, 1);

beta_belonging_betrayal ~ normal(0, 0.43); // 99% prob between -1 and 1

beta_belonging_wrongdoing ~ normal(0, 0.43);

beta_burden_wrongdoing ~ normal(0, 0.43);

beta_burden_betrayal ~ normal(0, 0.43);

beta_ideation_betrayal ~ normal(0, 0.43);

beta_ideation_wrongdoing ~ normal(0, 0.43);

beta_ideation_belonging ~ normal(0, 0.43);

beta_ideation_burden ~ normal(0, 0.43);

sigma_belonging ~ normal(0, 0.39); // 99% prob between 0 and 1

sigma_burden ~ normal(0, 0.39);

sigma_ideation ~ normal(0, 0.39);

for (n in 1:(N_items_betrayal)) {

pi_betrayal[n] ~ dirichlet(alpha_betrayal_wrongdoing);

}

for (n in 1:(N_items_wrongdoing)) {

pi_wrongdoing[n] ~ dirichlet(alpha_betrayal_wrongdoing);

}

for (n in 1:(N_items_belonging)) {

pi_belonging[n] ~ dirichlet(alpha_belonging_burden);

}

for (n in 1:(N_items_burden)) {

pi_burden[n] ~ dirichlet(alpha_belonging_burden);

}

for (n in 1:(N_items_si)) {

pi_si[n] ~ dirichlet(alpha_si);

}

b_sib ~ normal(0, 0.39); // 99% prob between 0 and 1

alpha_sib ~ normal(0, 1); // 99% prob density for bernoulli probit

// latent model – these are the modeled relationships among the latent variables

belonging ~ normal(beta_belonging_wrongdoing * wrongdoing + beta_belonging_betrayal * betrayal, sigma_belonging);

burden ~ normal(beta_burden_wrongdoing * wrongdoing + beta_burden_betrayal * betrayal, sigma_burden);

ideation ~ normal(beta_ideation_betrayal * betrayal + beta_ideation_wrongdoing * wrongdoing + beta_ideation_belonging * belonging + beta_ideation_burden * burden, sigma_ideation);

// observational model – modeling the observed Likert and binary responses for each item with the latent variables and cut-points

likert_betrayal ~ ordered_probit(betrayal[latent_i_betrayal], cutpoints_betrayal[item_i_betrayal]);

likert_wrongdoing ~ ordered_probit(wrongdoing[latent_i_wrongdoing], cutpoints_wrongdoing[item_i_wrongdoing]);

likert_belonging ~ ordered_probit(belonging[latent_i_belonging], cutpoints_belonging[item_i_belonging]);

likert_burden ~ ordered_probit(burden[latent_i_burden], cutpoints_burden[item_i_burden]);

likert_si ~ ordered_probit(ideation[latent_i_si], cutpoints_si[item_i_si]);

binary_si ~ bernoulli(Phi(alpha_sib + b_sib[item_i_sib] .* ideation[latent_i_sib]));

}

// the following block generates some summary statistics for the latent variables and makes predictions from the model for use in retrodictive checking

generated quantities {

real sd_betrayal = sd(betrayal);

real sd_wrongdoing = sd(wrongdoing);

real sd_belonging = sd(belonging);

real sd_burden = sd(burden);

real sd_ideation = sd(ideation);

real mu_betrayal = mean(betrayal);

real mu_wrongdoing = mean(wrongdoing);

real mu_belonging = mean(belonging);

real mu_burden = mean(burden);

real mu_ideation = mean(ideation);

vector[N_obs_betrayal] pred_betrayal;

vector[N_obs_wrongdoing] pred_wrongdoing;

vector[N_obs_belonging] pred_belonging;

vector[N_obs_burden] pred_burden;

vector[N_obs_si] pred_si;

vector[N_obs_sib] pred_sib;

for (n in 1:N_obs_betrayal) {

pred_betrayal[n] = ordered_probit_rng(betrayal[latent_i_betrayal[n]], cutpoints_betrayal[item_i_betrayal[n]]);

}

for (n in 1:N_obs_wrongdoing) {

pred_wrongdoing[n] = ordered_probit_rng(wrongdoing[latent_i_wrongdoing[n]], cutpoints_wrongdoing[item_i_wrongdoing[n]]);

}

for (n in 1:N_obs_belonging) {

pred_belonging[n] = ordered_probit_rng(belonging[latent_i_belonging[n]], cutpoints_belonging[item_i_belonging[n]]);

}

for (n in 1:N_obs_burden) {

pred_burden[n] = ordered_probit_rng(burden[latent_i_burden[n]], cutpoints_burden[item_i_burden[n]]);

}

for (n in 1:N_obs_si) {

pred_si[n] = ordered_probit_rng(ideation[latent_i_si[n]], cutpoints_si[item_i_si[n]]);

}

for (n in 1:N_obs_sib) {

pred_sib[n] = bernoulli_rng(Phi(alpha_sib + b_sib[item_i_sib[n]] * ideation[latent_i_sib[n]]));

}

}
